# Staged Paramedian Forehead Flap Reconstruction of a Large Oncologic Nasal Defect: A Case Report

**DOI:** 10.7759/cureus.90925

**Published:** 2025-08-25

**Authors:** José Alberto Fernandes, Bernardo Baptista, Ricardo Damaso, António Castanheira

**Affiliations:** 1 Department of Otorhinolaryngology, Unidade Local de Saúde Trás-os-Montes e Alto Douro, Vila Real, PRT; 2 Department of Otorhinolaryngology, Unidade Local de Saúde de São João, Porto, PRT

**Keywords:** auricular cartilage graft, facial plastic surgery, interpolated flap, nasal reconstruction, oncologic nasal defect, paramedian forehead flap, supratrochlear artery

## Abstract

Nasal reconstruction after oncologic resection presents a significant surgical challenge, particularly for defects exceeding 1.5 cm, which frequently require interpolated flaps, such as the paramedian forehead flap (PFF), given the need to restore both form and function. The PFF, a historical workhorse in nasal reconstruction, derives its reliability from an axial pedicle based on the supratrochlear artery, providing robust perfusion and an excellent skin match for large and complex nasal defects. We report the case of an 81-year-old male with basal cell carcinoma of the right nasal ala. Wide local excision resulted in a full-thickness defect involving external skin, structural cartilage, and internal lining. A staged reconstruction was performed. In the first stage, a vertically oriented PFF based on the right supratrochlear artery was elevated. Auricular cartilage was harvested to restore the nasal framework, while local mucosal advancement flaps reconstituted the internal lining. The PFF was then inset to reconstruct the external skin. Pedicle division and flap refinement were carried out three weeks later. The postoperative course was uneventful. At six-month follow-up, the patient demonstrated excellent nasal contour, preserved airway function, and no evidence of recurrence. Donor-site morbidity was minimal and well tolerated. Reconstruction successfully restored all three layers of the nasal wall, with high satisfaction reported by both the patient and the surgical team. This case supports the role of the PFF as a workhorse for multilayer nasal reconstruction, yielding predictable outcomes with favorable aesthetic integration.

## Introduction

Reconstruction of nasal and midfacial defects remains one of the most technically demanding and aesthetically sensitive challenges in facial plastic and reconstructive surgery. Owing to the nose's central anatomical location and its essential roles in respiration, olfaction, and facial symmetry, precise restoration of both form and function is critical. Beyond functional impairment, major nasal defects carry a substantial psychosocial burden, causing significant psychological distress and social inhibition and adversely affecting self-image, social functioning, and overall quality of life.

Successful reconstruction requires not only surgical expertise but also a patient-centered approach that accounts for defect size, depth, and subunit involvement, as well as patient-specific factors such as age, comorbidities, and prior treatments. For extensive defects, particularly those resulting from oncologic resections, the objective extends beyond closure to comprehensive restoration of nasal architecture, preserving function while achieving aesthetically acceptable outcomes.

Three-layer nasal reconstruction requires simultaneous restoration of (i) a well-vascularized internal lining that resists contraction and preserves airway patency; (ii) a stable cartilaginous framework to maintain projection, alar rim support, and valve competence; and (iii) an external skin envelope that matches adjacent subunits in thickness, color, and contour. Failure to address each layer systematically can precipitate functional compromise, most notably nasal valve collapse, intranasal stenosis, and airway obstruction, as well as aesthetic failure, including contour irregularities, alar retraction, and flap contracture.

The paramedian forehead flap (PFF) is widely regarded as the gold standard for external nasal cutaneous coverage in large and complex defects. Its reliability derives from a consistent axial blood supply via the supratrochlear artery, ensuring robust perfusion even in patients with comorbidities or compromised vascularity [1,2]. The PFF also provides exceptional design flexibility, allowing precise adaptation to the three-dimensional contours of the nasal framework. When used in a staged fashion, typically in two or three stages, it permits accurate inset, progressive thinning, and contour refinement in accordance with aesthetic subunit principles. Importantly, in multilayer defects, the PFF provides the external skin envelope and is combined with separate reconstructive strategies for internal lining and structural support.

The forehead skin's close match to nasal skin in terms of thickness, texture, and color further enhances aesthetic outcomes, particularly in the reconstruction of the tip or ala, where even subtle discrepancies can significantly affect cosmesis [3,4]. In oncologic cases, where full-thickness resections are common, the PFF's robust vascularity supports cartilage graft survival and tolerates staged refinements, making it especially well-suited for comprehensive nasal reconstruction.

Owing to its long clinical history, consistent refinements, and reliable outcomes, the PFF remains an indispensable tool in modern nasal reconstruction, offering durable coverage and favorable aesthetic integration when combined with appropriate techniques for lining and framework restoration [5].

## Case presentation

An 81-year-old male presented with an 18 × 14 mm cutaneous lesion on the right nasal ala. Histopathological examination confirmed basal cell carcinoma, a nodular-infiltrative subtype, without perineural or lymphovascular invasion. Wide local excision created a 28 × 24 mm full-thickness alar defect involving external skin, cartilage framework, and internal lining. Margins were negative: the closest peripheral margin measured 15 mm, and the deep margin was clear. The specimen demonstrated focal perichondrial and superficial cartilaginous invasion. Pathologic staging was pT1 (AJCC 8th edition), and no adjuvant therapy was indicated.

**Figure 1:**
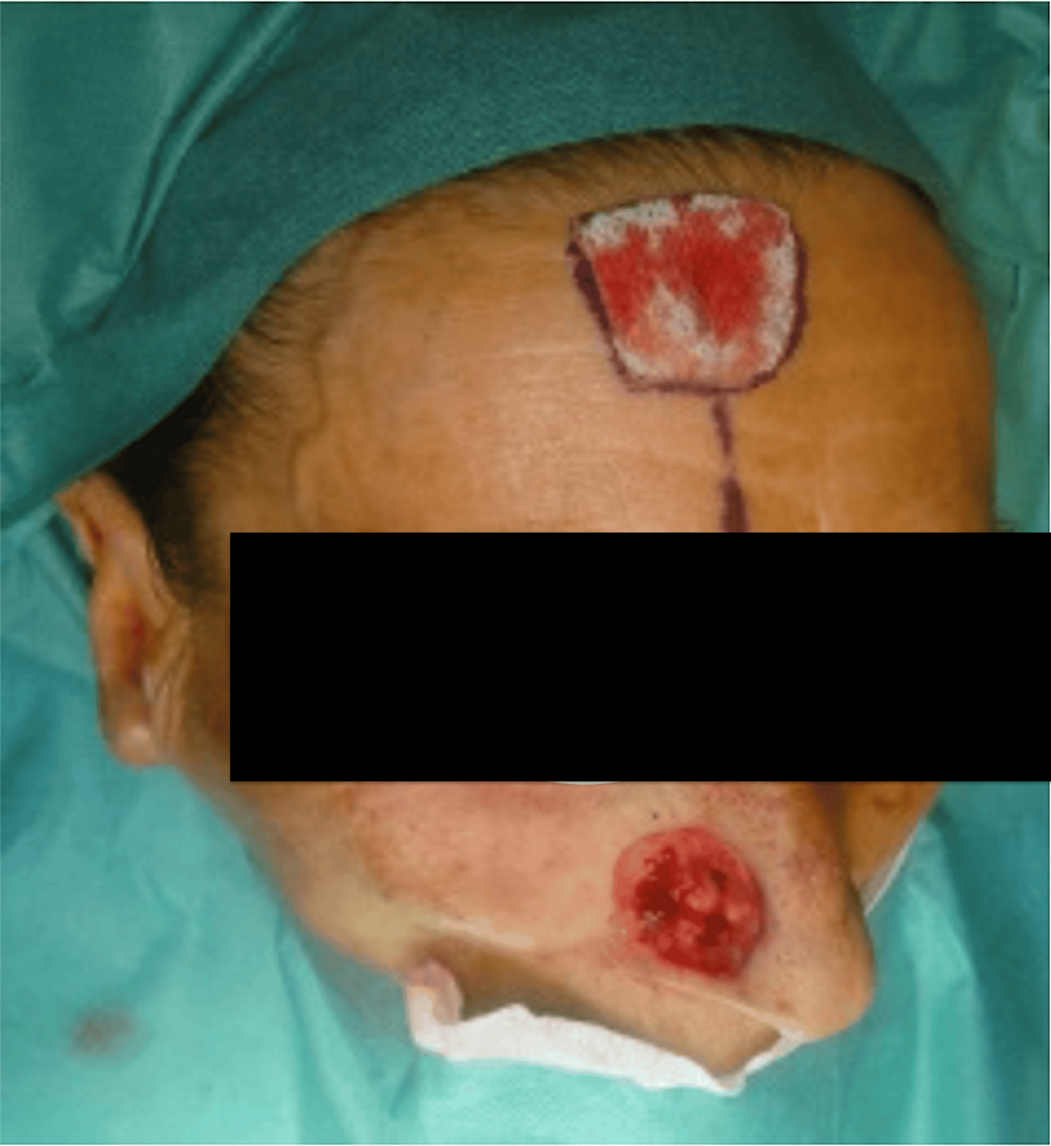
Design of the vertically oriented paramedian forehead flap based on the right supratrochlear artery. A full-thickness nasal defect is visible following wide local excision of a basal cell carcinoma

Reconstruction was planned in a two-stage fashion. The ipsilateral supratrochlear artery was mapped with a handheld Doppler, which confirmed a robust arterial signal at the intended pivot point, approximately 1.7 cm from the midline. A vertical PFF was designed as an ellipse based on this pedicle, with a width of 10-12 mm to optimize inflow and outflow, and a pivot just above the medial brow to allow a tension-free arc of rotation. The skin paddle was sized to a sterile template of the defect with modest overlength to permit inset and later thinning. Elevation proceeded in the subgaleal plane, preserving the supratrochlear pedicle and distal subdermal plexus. Hemostasis was achieved with bipolar cautery, and the pedicle was protected with moist gauze throughout the procedure (Figure 1).

**Figure 2:**
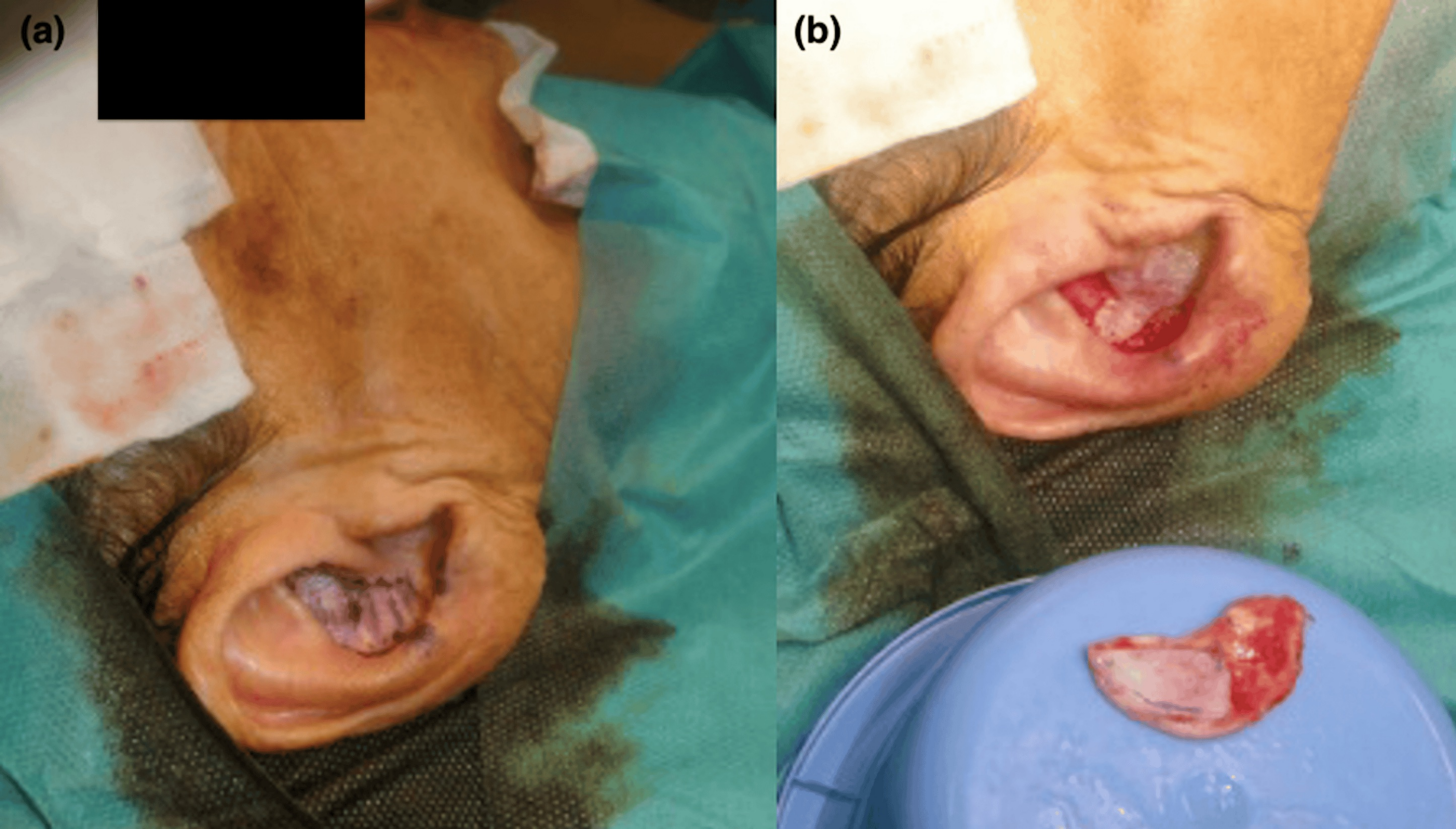
(a) Incision over the ipsilateral auricular concha for harvesting an autologous cartilage graft. (b) Conchal cartilage graft utilizing its natural curvature to replicate the native alar contour and provide structural support

The internal lining was reconstructed using local mucosal advancement flaps from adjacent vestibular tissue. Structural support was restored with autologous auricular conchal cartilage harvested from the ipsilateral ear (Figure 2).

**Figure 3:**
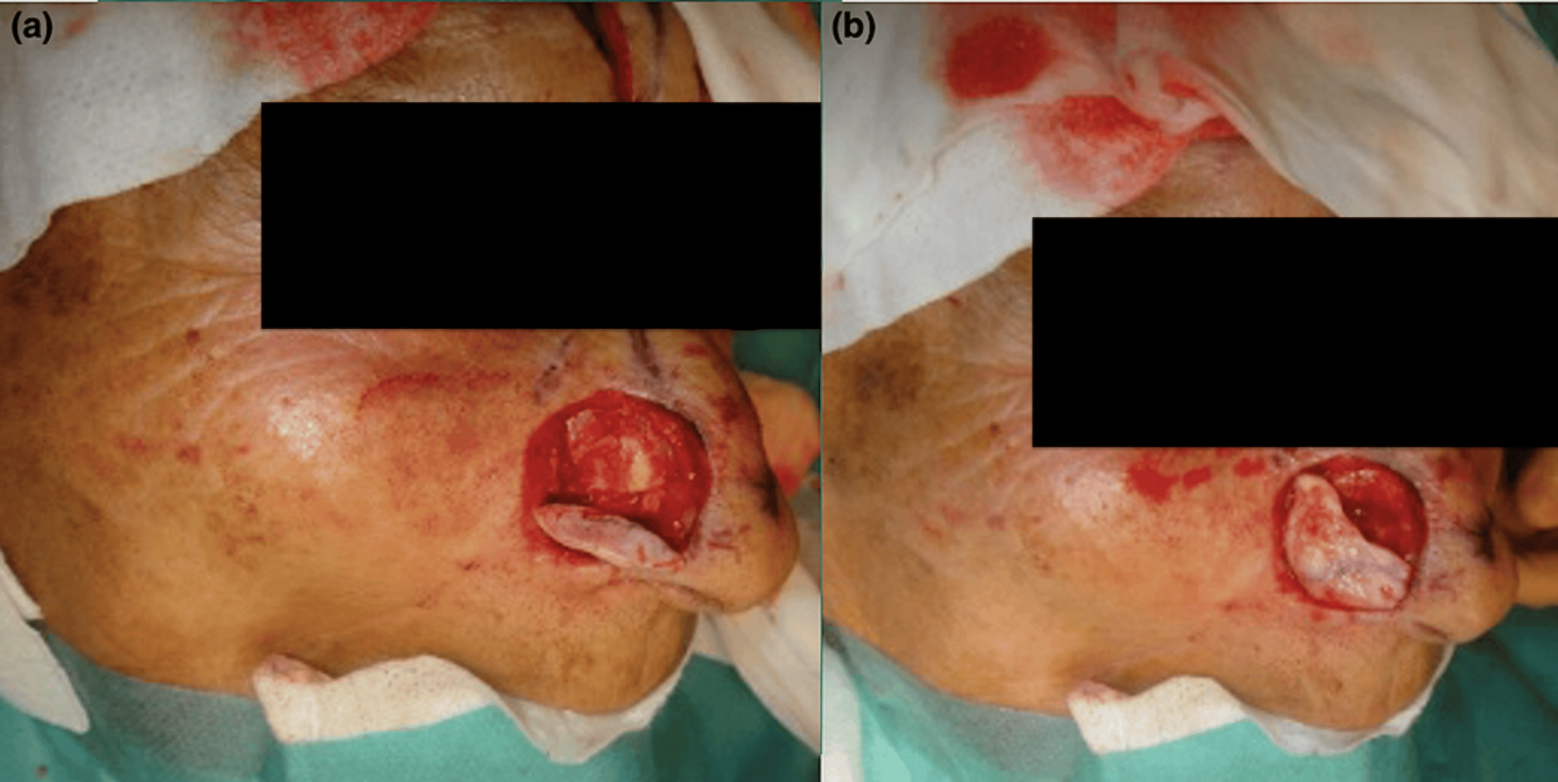
(a) Placement of the conchal cartilage graft, contoured to the defect, to restore alar contour and provide structural support. (b) Graft positioned within the defect, demonstrating conformity with the native alar framework

The natural curvature of the concha closely matched the convexity of the native ala and required only minor edge beveling. Orientation was preserved: the convex surface recreated the external contour, while the concave surface faced the vestibule. The graft was fixated with interrupted 5-0 PDS sutures to maintain alar contour and valve competence (Figure 3).

**Figure 4:**
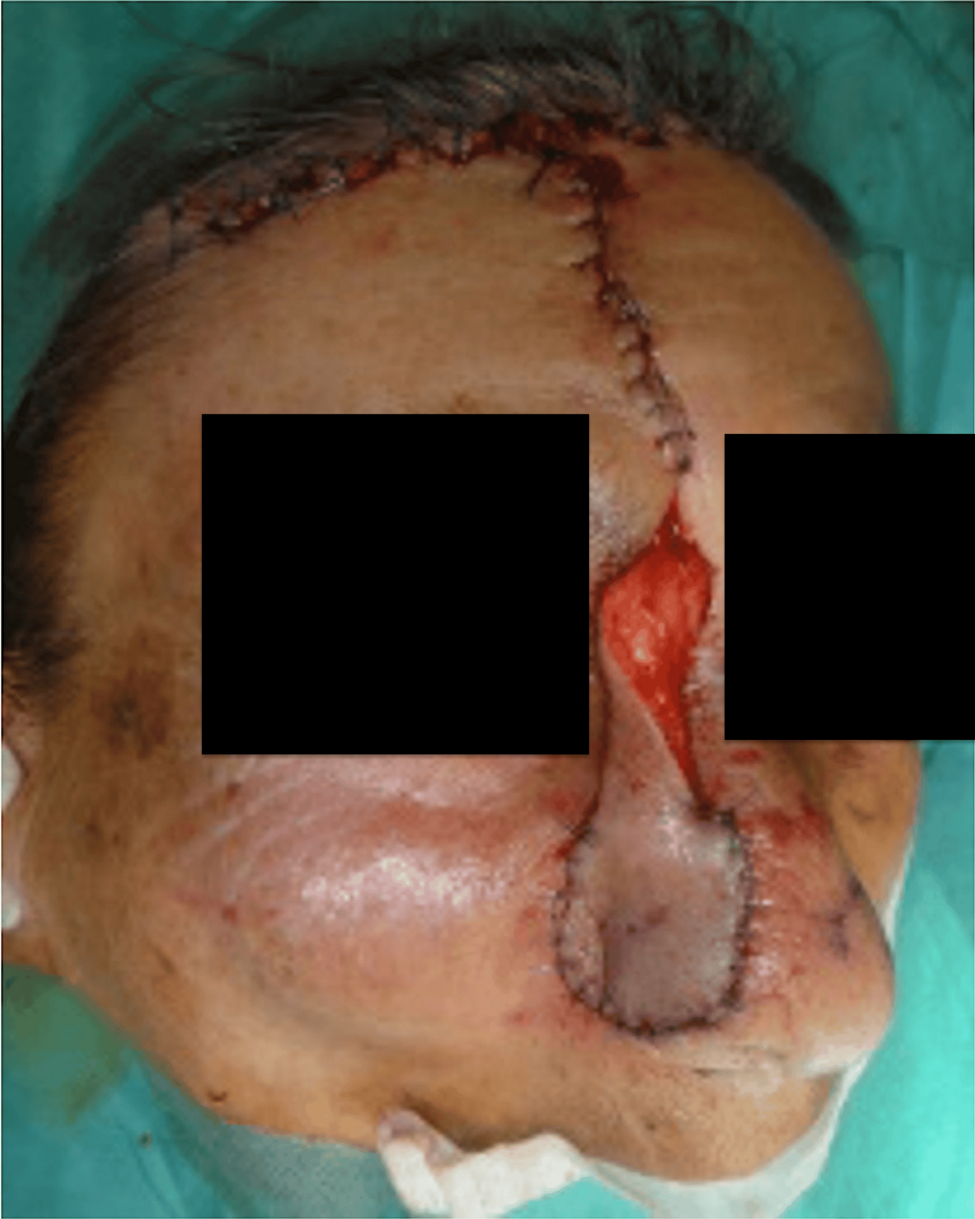
Transposition of the interpolated PFF to the nasal defect with preservation of the axial pedicle PFF: paramedian forehead flap

The flap was advanced and inset without tension using interrupted 5-0 Vicryl® dermal sutures and a running 6-0 nylon epidermal closure. Anchoring stitches at the alar rim and soft triangle aligned the flap with aesthetic subunit borders. Limited distal thinning and edge beveling refined the contour, and quilting sutures were placed selectively to obliterate dead space and recreate the alar-facial groove, as well as to smooth the dorsum-tip transition. The forehead donor site was closed primarily after meticulous hemostasis (Figure 4).

**Figure 5:**
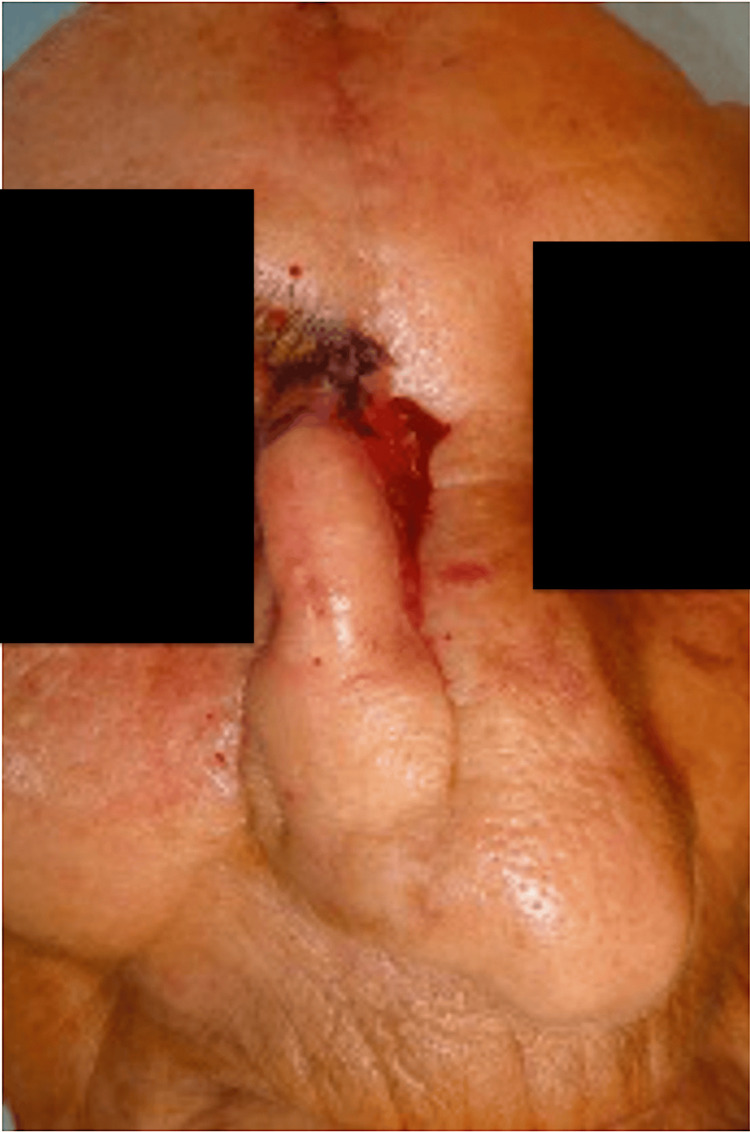
Immediate postoperative view following pedicle division at three weeks, with refinement of flap contour and final insetting

Pedicle division was performed on postoperative day 21. At division, targeted refinements included distal flap thinning to match alar skin thickness, edge beveling to re-establish the alar-facial groove and soft triangle, limited repositioning of the conchal graft to refine the alar rim, selective quilting to smooth the dorsum-tip transition, and minor scar revision to optimize color and texture blending (Figure 5).

**Figure 6:**
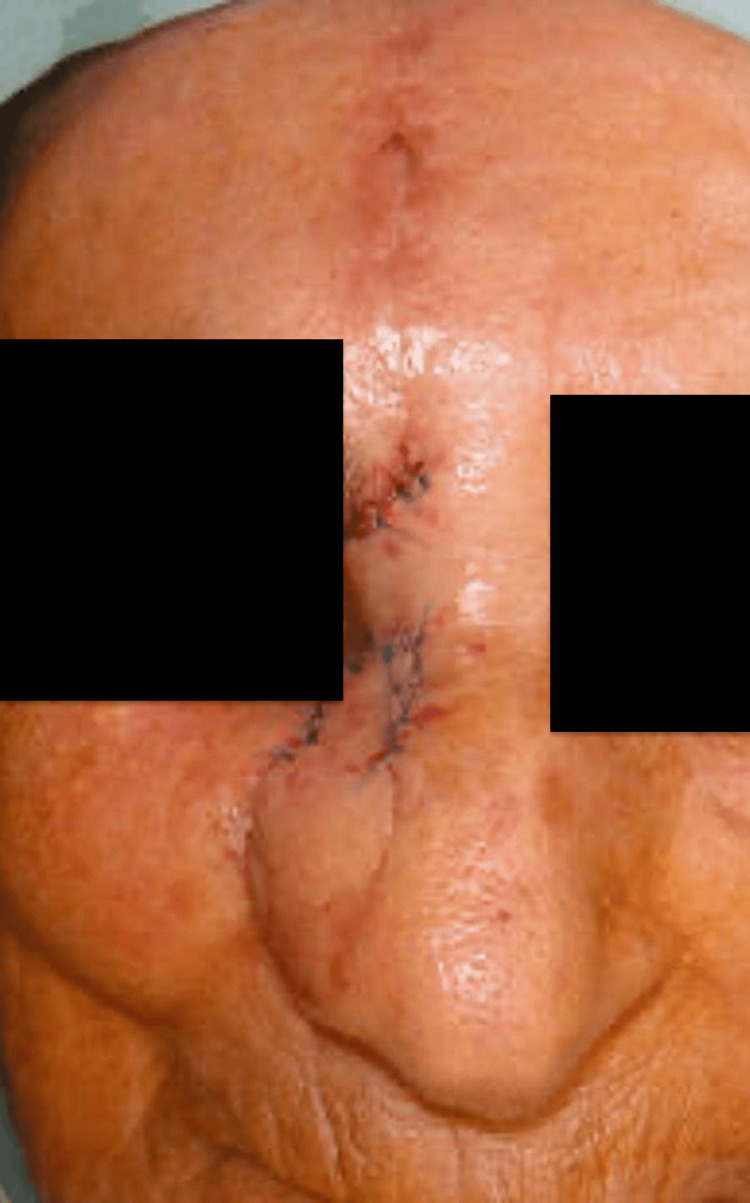
Intermediate postoperative outcome showing satisfactory integration of the flap and maintenance of nasal airway patency

Postoperative care included pedicle protection with a light bolster and instructions to avoid pressure and cold exposure. Nasal care consisted of humidification, saline irrigations, and topical ointment applied to suture lines. Follow-up visits on days 7, 14, and 21 confirmed flap viability, airway patency, and wound healing. Sutures were removed on day 14. No complications such as hematoma, necrosis, or infection were observed (Figure 6).

**Figure 7:**
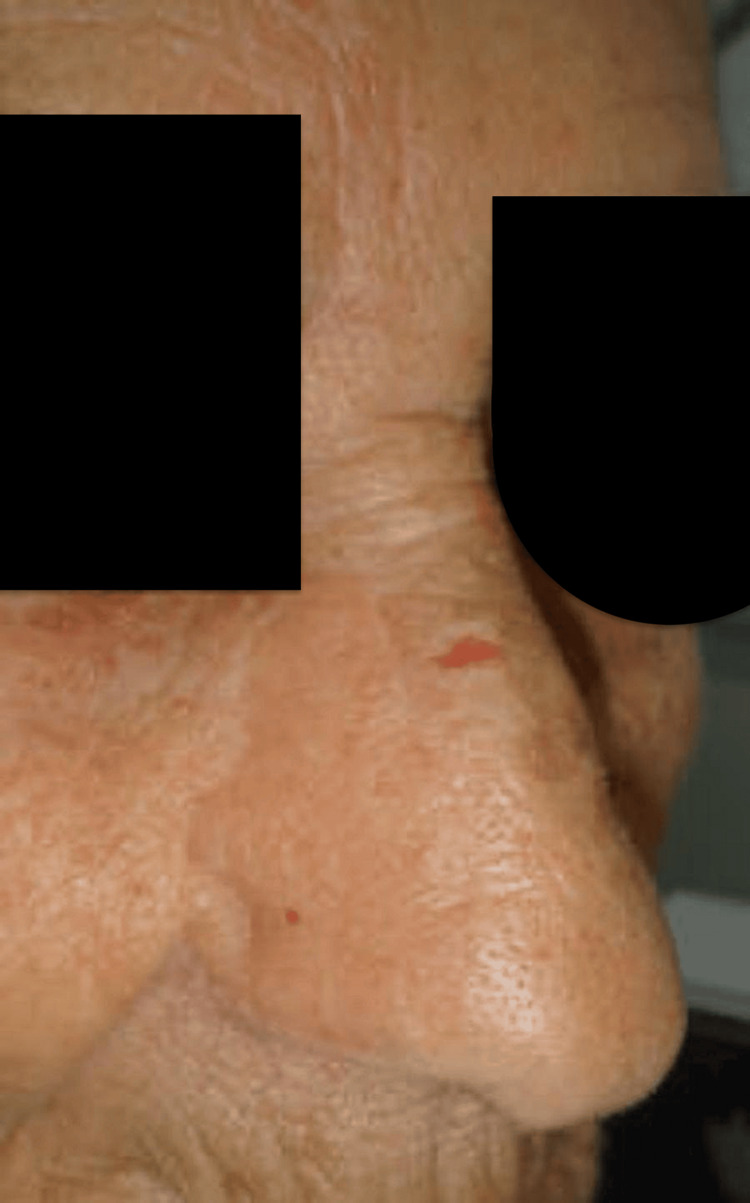
The final postoperative result at six months demonstrating excellent nasal contour, color, and texture match, with minimal and acceptable forehead donor-site scarring

At six-month follow-up, the patient demonstrated excellent nasal contour, preserved airway patency, and no evidence of recurrence. The reconstructed ala showed harmonious skin tone and texture, with minimal donor-site morbidity and an acceptable forehead scar. Both functional and aesthetic outcomes were judged highly satisfactory by the patient and surgical team (Figure 7).

## Discussion

The successful outcome in this case aligns with existing literature confirming the PFF as a reliable and versatile option for complex nasal reconstruction [2-6]. In our patient, who had a three-layer right alar defect measuring 2.5 × 2.0 cm with perichondrial and superficial cartilaginous involvement, the PFF provided dependable axial perfusion and an excellent skin match. These advantages make it preferable to local options such as bilobed, dorsal nasal, or nasolabial flaps, which are limited by defect size, thickness mismatch, and risk of alar retraction when defects exceed ~1.5 cm or involve multiple layers [1-6].

Our case, which proceeded without complications, supports the low morbidity profile reported in large PFF series, such as that of Chen et al., and likely reflects meticulous preservation of the axial pedicle, Doppler-guided flap design, and staged inset with pedicle division at approximately three weeks [7,8]. This mirrors outcomes from contemporary cohorts, where adverse events are uncommon and generally manageable when technical safeguards are observed [9,10].

Key intraoperative choices were consistent with principles emphasized in authoritative references. Ipsilateral conchal cartilage was harvested to exploit its natural curvature, requiring only minimal edge beveling. Orientation was preserved, with the convex surface facing outward to replicate the alar contour and the concave surface facing the vestibule to maintain valve competence [3,5]. Preoperative Doppler mapping confirmed a robust supratrochlear signal, ~1.7 cm lateral to the midline, which guided flap side selection, pedicle width, and arc of rotation to ensure a tension-free inset and dependable perfusion [2,11,12]. Our decision to divide the pedicle at three weeks reflected both standard practice and patient-specific perfusion findings (brisk capillary refill, uniform warmth, no venous congestion). This cautious interval likely facilitated cartilage graft survival and minimized contour trade-offs during staged thinning [6,8].

At six months, the reconstruction maintained alar contour and valve competence without notching, bulk, or trapdoor deformity. The color and texture of the forehead skin blended favorably with the surrounding subunits. These results are consistent with published expectations for staged PFF in multilayer alar reconstruction and comparable to large series reporting reliable functional preservation and high aesthetic satisfaction [7,8,13].

Recent reports of the "dragonfly flap," a single-stage modification that incorporates forehead skin and the frontalis muscle for lining, aim to reduce the need for staging in subtotal or near-total nasal defects. However, early reports emphasize feasibility rather than comparative outcomes, and concerns remain regarding intranasal bulk, mucosalization of muscle, and donor-site morbidity [14]. In our case, which required precise, thin lining and staged contouring for a segmental alar defect, the conventional PFF was preferable. The staged approach offered opportunities for refinement and minimized airway compromise, advantages not yet demonstrated for the dragonfly variant. Broader validation and long-term functional data will be required before this alternative can be considered equivalent to the established technique.

This report is limited by its single-patient design, which restricts generalizability across broader populations with diverse anatomical and pathological characteristics. The six-month follow-up period is adequate to document early healing and initial cosmetic results but insufficient to assess long-term outcomes, such as final scar maturation, late contracture, and the stability of the cartilage graft. Outcome assessment relied on clinical examination and standard photography, without the use of validated patient-reported outcome measures (PROMs) or objective functional testing. Future cases at our institution will incorporate standardized PROMs, including the Nasal Obstruction Symptom Evaluation (NOSE) scale and relevant FACE-Q modules, collected at defined intervals (3, 6, 12, and ≥24 months). Additional limitations include potential selection bias, the experience of a single surgeon, the specific defect topology and skin type, and the absence of objective perfusion imaging (e.g., indocyanine green angiography) that could have informed intraoperative and staging decisions.

## Conclusions

The PFF remains the gold standard for complex nasal reconstruction, particularly for defects larger than 1.5-2.0 cm where local flaps or primary closure are not feasible. It offers reliable vascularity, excellent skin match, and durable functional and aesthetic outcomes. In addition to these advantages, its harvest is straightforward, guided by consistent anatomic landmarks and a robust axial pedicle, making the technique reproducible and dependable without being excessively technically demanding.
